# Higher n3-fatty acid status is associated with lower risk of iron depletion among food insecure Canadian Inuit women

**DOI:** 10.1186/1471-2458-13-289

**Published:** 2013-04-02

**Authors:** Jennifer A Jamieson, Harriet V Kuhnlein, Hope A Weiler, Grace M Egeland

**Affiliations:** 1 Centre for Indigenous Peoples’ Nutrition and Environment (CINE), McGill University, Montreal, Canada; 2 School of Dietetics and Human Nutrition, McGill University, Montreal, Canada; 3 Department of Public Health and Primary Health Care, University of Bergen and The Norwegian Institute of Public Health, Bergen, Norway

## Abstract

**Background:**

High rates of iron deficiency and anemia are common among Inuit and Arctic women despite a traditional diet based on animal source foods. However, representative data on iron status and relevant determinants for this population are lacking. The objectives were to determine the prevalence of anemia and depletion of iron stores, then to identify correlates of iron status in non-pregnant Canadian Inuit women.

**Methods:**

In a cross-sectional survey of 1550 women in the International Polar Year Inuit Health Survey, 2007-2008, hemoglobin, serum ferritin, soluble transferrin receptor (on a subset), C-reactive protein (CRP), RBC fatty acid composition, and *H pylori* serology were analyzed on fasting venous blood. Sociodemographic, food security status, anthropometric, dietary, and health data were collected. Correlates of iron status were assessed with multivariate linear and logistic models.

**Results:**

Anemia was observed in 21.7% and iron deficient erythropoiesis in 3.3% of women. For women with CRP ≤ 10 mg/L (n = 1260) 29.4% had depleted iron stores. Inadequate iron intakes were observed in 16% of premenopausal and <1% of postmenopausal women. Among food insecure women, higher long-chain (n-3) polyunsaturated fatty acid (LC-PUFA) status, which reflects a more traditional food pattern, was associated with reduced risk of iron depletion.

**Conclusions:**

Iron depletion and anemia are a concern for Inuit women despite adequate total dietary iron intake primarily from heme sources. The high prevalence of *H. pylori* exposure, together with dietary iron adequacy, suggests an inflammation-driven iron deficiency and mild anemia. The anti-inflammatory properties of LC-PUFA may be important for iron status in this population.

## Background

Iron deficiency (ID) is the most common nutritional deficiency in the world and one of the leading 15 contributors to the global burden of disease [1]. Although adequacy of dietary iron intake has been observed among Arctic Indigenous Peoples [2-4] moderate to high rates of both ID (22-39%) and anemia (10-42%) have been reported for Canadian Inuit [4-6] and Alaska Native [3] women. In general, higher rates of ID have been observed in pre-menopausal women, while anemia rates have been greater in postmenopausal women [7]. Changes in lifestyle and dietary patterns over recent decades for Arctic Indigenous Peoples may contribute to this phenomenon. Animal-source foods, central to the traditional Inuit diet, are rich sources of bioavailable iron [8]. Less reliance upon traditional food (TF), a well documented feature of the nutrition transition, is most evident in younger Inuit [8,9], and therefore of particular relevance to women of childbearing years. Whether reduced TF consumption contributes to low iron status indices among Inuit women or if other endemic factors may account for iron depletion has not recently been thoroughly investigated. However, data from a 1993-94 Greenlandic Inuit survey (n = 224) suggested that iron depletion was over twice as common and iron overload one-tenth as prevalent among women in a westernized community compared to a more traditional community [10]. Therefore declining use of TF may place Inuit women at increased risk of ID and anemia.

The extent to which iron deficiency anemia (IDA) can currently explain anemia prevalence for adult Inuit women, given the ongoing nutrition transition, requires clarification. In the Northwest Territories, Canada (1972) and Greenland (1993-94), IDA rates were <4% among women despite anemia rates of 10-18% [6,11]. More recently, in Nunavik (2004), 21% of women were classified with IDA, with 43% anemic overall [12]. Other factors endemic to the Arctic and with particular relevance to iron status may include acute or chronic inflammation and obesity. Obesity among Inuit is associated with indicators of acculturation and higher socioeconomic status [13]. The chronic low-grade inflammation of obesity is linked with hepcidin synthesis and therefore decreased iron absorption and iron stores [14,15]. Chronic infection with *Helicobacter pylori* could also contribute to inflammation [3]. Conversely, the high n3-PUFA intakes associated with traditional dietary patterns for Inuit [16] may protect against inflammation and support iron status.

The objectives of this study were to determine the prevalence of anemia and depletion of iron stores, then to identify correlates of iron status in Canadian Inuit women of Inuvialuit Settlement Region (ISR), Nunavut Territory, and Nunatsiavut of Northern Labrador.

## Methods

### Study population

The studied population was non-pregnant, self-identified Inuk women, 18 years of age or older from ISR, Nunavut, and Nunatsiavut in Arctic Canada. According to the 2006 Statistics Canada Census the Inuit population was 50, 485.

### Sampling and data collection

A cross-sectional Inuit health survey of adults residing in randomly selected households was carried out between August to September, 2007, and August to October, 2008 in 36 Canadian Arctic communities [17]. Households were randomly selected from a list of house numbers in each participating community. Of the 2796 Inuit households approached, 1901 households (68%) agreed to participate, with a total of 2595 adult participants, of whom 61.5% were women. Forty-five women were excluded from current analyses due to possible pregnancy or lactation. After providing voluntary and informed consent, a 24-hour recall, semi-quantitative food frequency questionnaire, 5 general questionnaires, a fasting venous blood sample, and anthropometric measurements were obtained either on-land or utilizing the Canadian Coast Guard Ship Amundsen. Venous blood samples were obtained from 1403 (87.9%) female participants of whom 1309 women had both SF and high-sensitivity C-reactive protein (hs-CRP) measured and met the inclusion criteria. Appropriate regional research licenses were obtained and ethical approval for the survey was granted from the McGill University Faculty of Medicine Institutional Review Board. Survey design and implementation was directed by a participatory process [17].

### Clinical assessment

Weight and body composition were measured using bioelectrical impedance analysis (Tanita TBF-300GS, Arlington Heights, IL, USA) and height was measured with a portable stadiometer (Road Rod 214, Seca, Maryland). Normal weight, overweight and obesity were defined by the WHO classification system [18]. Percent body fat was classified according to the manufacturer’s age-appropriate healthy body fat ranges. For women, an at-risk body fat percentage was defined as >33%, >34%, and >36% for ages 18-39, 40-59, and 60-99 y, respectively.

### Laboratory analyses

Participants were requested to fast at least 8 hours overnight, prior to morning venous blood collection. Hemoglobin measures were obtained from venous blood drops or blood drops from a finger prick using the azidemethemoglobin method with HemoCue™ 201+ portable photometer (HemoCue, Inc., Lake Forest, California), and adjusted for cigarette smoking according to WHO guidelines [19]. Prevalence estimates of anemia were derived from venous samples only and categorized according to the WHO 120 g/L cut-off for non-pregnant women [19]. Serum samples were analyzed for ferritin (automated chemiluminescence assay; Liaison Ferritin;Diasorin, Italy), hs-CRP (auto-analyzer; Beckman Coulter, Brea, CA, USA), and soluble transferrin receptor (sTfR) concentration on a subsample (n = 652, ELISA assay; R&D Systems, Minneapolis, USA). Serum sTfR > 2.75 mg/L was defined as iron deficient erythropoiesis (IDE), as suggested by the manufacturer. Depleted iron stores was defined by SF < 15 μg/L or SF = 15-50 μg/L in the presence of acute inflammation (hs-CRP ≥10 mg/L). SF > 200 μg/L in the absence of acute inflammation was used to define elevated iron stores in order to compare across studies of Inuit populations [12] with sex and age-appropriate cut-offs for iron overload [20] also determined. Iron deficiency anemia (IDA) was defined as anemia + SF <15 μg/L or SF 15-50 μg/L + hs-CRP > 10 mg/L. Immunoenzymatic methods (ELISA) were used to detect IgG antibodies against *H. pylori* in serum (Calbiotech; Spring Valley,CA, USA) and RBC membranes were analyzed for fatty acid composition (Lipid Analytical Laboratories Inc., University of Guelph Research Park, Guelph, ON), as previously described [21]. Total EPA and DHA were expressed as % of total fatty acids and hereafter referred to as LC-PUFA (long chain-PUFA).

### Dietary assessment

Dietary assessment was conducted by trained interviewers using a single 24 hour recall with a four stage, multi-pass approach [22] and a 42-item semi-quantitative food frequency questionnaire. Portion sizes were estimated with a graduated, three-dimensional food model kit (Santé Québec). Recall data were entered into CANDAT software (Godin London Inc,. London, Ontario, Canada) and nutrient analyses obtained from the 2007b Canadian Nutrient File (CNF) and additional databases as described previously [21]. There were no missing nutrient values in the analysis. Recall data were available for 1248 female participants after 25 recalls were excluded due to incompleteness. Dietary iron adequacy was assessed by the Dietary Reference Intake (DRI) probability method (pre-menopausal women) or the Estimated Average Requirement (EAR) cut-point method (post-menopausal women) using the SIDE method [23] and SIDE software [24] in which within-subject variation estimates for iron intake were obtained from dietary surveys with Canadian Inuit populations [9]. Intake of iron and dietary enhancers and inhibitors of iron were analyzed for women using 24-hour recall data. Twenty-nine 18 year olds (with different DRI requirements for their age group) were excluded from this intake analysis. Reported food items were classified as TF (derived from hunting, fishing or gathering) or market foods (imported foods available for purchase). Nutritional supplement and medication use was recorded by a nurse. Supplement content was not included in nutrient analysis of the 24 hour recalls as the majority of supplement users could not recall the brand or amount of supplement taken. TF frequency data were available for 1229 female participants. Frequency of TF use over the past 12 months was documented for in-season and off-season consumption of each item. Seasons were established according to locally developed regional wildlife harvest calendars and intakes adjusted to frequency per month (assuming 30.4 days per month).

### Questionnaires

Questionnaires for sociodemographic, health and household characteristics were adapted from Greenlandic and Nunavik (Canada) Inuit Health Surveys [25] and the Aboriginal Peoples Survey [26], The household questionnaire included a version of the 18-item USDA Household Food Security Survey Module [27], with details of the questionnaire and classification of household food security described elsewhere [28]. A household food security score was dichotomized into secure or insecure and applied to each adult household member for analyses. Current smoking status was assessed as yes or no and quantified by cigarettes per day. Menopausal status was self-reported and defined by absence of a regular menstrual period or age ≥50 y if menstrual status not reported. Women were asked about contraceptive use to account for known effects of hormonal contraceptives (estrogen and/or progesterone) and intrauterine device use on iron status [29].

### Statistical analysis

SF and sTfR concentrations were log_10_ transformed to improve normality of the respective distributions. Weighted prevalence estimates of iron status are provided with 95% confidence intervals. Sampling weights reflected the proportion of participating women using Statistics Canada’s Census data of age-appropriate Inuit women by community. Age categories for prevalence estimates were based on the DRI recommendations for iron intake [30], although age-groups 51-70 and ≥70 were combined due to small sample size among the elderly. Independent determinants of depleted iron stores vs adequate iron stores were assessed with a multivariable logistic model, with community included as a random effect to improve model fit. *A priori* selection of variables known or suspected to be related to iron status were evaluated. The model was based on 1062 women with SF available and hs-CRP ≤ 10 mg/L to reduce invalid SF results due to inflammation. Low-grade inflammation (hs-CRP 3-10 mg/L) was included as a control variable. Sample size limited interaction testing to only 2x2 interactions between main effects. Model specification was verified with STATA linktest and standard model diagnostics were performed. Generalized linear models were used to investigate the relationship between LC-PUFA status and obesity. All analyses were performed in STATA (version 11; StataCorp LP, College Station, TX). *P* values were all two-sided and significance was set at *P* ≤ 0.05, with adjustments for multiple comparisons as appropriate.

## Results

### Study population

Mean age ± SD of the study population was 42 ± 15 y (range: 18-90 y), with 25% of women overweight and 43% obese using WHO criteria. Underweight was infrequent (1%). Seventy-one percent of women currently smoked, with a median 10 cigarettes/day (IQR: 6-12). Prescription or non-prescription medication use was reported by 43% of women, oral or hormonal contraceptives were used by 11% of premenopausal women and intrauterine device by 4%. In all, 70% of women were classified as premenopausal. High blood pressure, diabetes mellitus, and high cholesterol were self-reported in 28%, 7%, and 12% of women respectively and a low hs-CRP concentration (<3 mg/L) was observed in 71% of female participants. Sixty-two women reported taking an iron-containing supplement and were included in analyses because of lack of difference in SF between supplement users and non-users. TF consumption was reported by 57.3% of women on the day prior to the survey, representing 6.8% of energy intake. The majority of the sample (61%) was classified as married and 66% reported living with an active hunter in the home.

### Iron status and anemia

**Figure 1:**
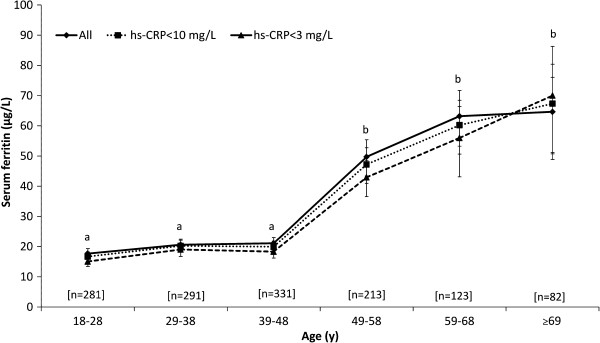
**Serum ferritin concentrations of Inuit women by hs-CRP status and age-group.** Values are geometric means (95% confidence interval). For unadjusted ferritin, *n* are shown on the X axis. Data points without a common letter differ between age-groups for all women. Excluding women with elevated or high CRP did not affect the interpretation of statistical differences.

Prevalence of anemia was moderate and similar across all age-groups (Table 1). IDA accounted for approximately half of the anemia cases, with the majority of IDA cases observed in premenopausal women. There was little evidence of iron deficient erythropoiesis (Table 1) but depleted iron stores were common and 74.5% of the population had SF < 50 μg/L. Elevated iron stores were rare and severe iron overload (SF > 700 μg/L) was absent. When using age-appropriate cut-offs for iron overload [20] rates were 0.3% (18-30 y), 0.9% (31-50 y) and 4.6% (≥51 y). SF concentration was low and stable in women until after age 48 (Figure 1) when it was more than 2-fold higher, whereas sTfR was low in all age-groups (Table 2). Adjusting for CRP < 10 mg/L and CRP < 3 did not appreciably alter SF concentrations or prevalence of iron depletion.

**Table 1:** **Weighted prevalence of iron status and anemia among Inuit women by age group**^**1**^

**Age, y**	**Anemia**^**2**^		**Depleted iron stores**		**Elevated iron stores**		**Iron deficiency anemia**		**Iron deficient erthrypoiesis**^**3**^		**Iron intake < EAR**^**4**^	
	***n***	***% *(95% CI)**	***n***	***% *(95% CI)**	***n***	***% *(95% CI)**	***n***	***% *(95% CI)**	***n***	***% *(95% CI)**	***n***	***%***
18-30	169	17.9	333	40.3	333	-	166	11.7	153	1.0	312	15.7
		(12.7-24.6)		(34.3-46.6)				(7.4-18.2)		(0.1-6.9)		
31-50	326	21.3	583	37.0	583	0.9	316	15.4	294	6.2	583	17.3
		(16.7-26.8)		(32.7-41.6)		(0.3-3.0)		(11.5-20.3)		(3.3-11.4)		
≥51	202	24.9	344	9.2	344	5.9	182	3.9	186	0.7	326	0.6
		(18.1-33.3)		(6.3-13.2)		(3.4-10.1)		(1.5-9.6)		(0.2-3.0)		
Total	697	21.7	1260	29.4	1260	2.2	664	11.1	633	3.3	-	-
		(18.3-25.5)		(26.7-32.3)		(1.3-3.6)		(8.7-14.0)		(1.9-5.8)		

^1^ Where anemia = hemoglobin < 120 g/L; depleted iron stores = serum ferritin <15 μg/L or ferritin 15-50 μg/L + CRP > 10 mg/L; elevated iron stores = ferritin > 200 μg/L + CRP ≤ 10 mg/L; iron deficiency anemia = depleted iron stores + anemic; iron deficient erythropoiesis = serum soluble transferrin receptor (sTfR) > 2.75 mg/L. EAR = estimated average requirement.

^2^Analyses on a subset with venous blood sampling for hemoglobin determination.

^3^Analyses on a subset with soluble serum transferrin receptor measurements.

^4^Analyses on adjusted intakes for women aged 19-50 y using the probability method and women aged > 50 y using the EAR cut-point method, with women aged 18 y excluded.

**Table 2:** **Associations between serum ferritin and dietary and health-related variables for Inuit women**^1^

**Dietary intake on the previous day, *unit***	***n***		**Multivariate**	**P-value**
			**Coefficient**^**3**^	
Energy, *MJ*	1123	7.56 (5.43-10.4)		
Traditional food,% *energy*	1123	6.8 (0-24)	0.001	0.008
Traditional food meat, *g*	1123	81.6 (0-277)		
All meat, *g*	1123	235 (128-420)		
Dietary iron, *mg*	1123	12.8 (8.2-20.3)		
Dietary iron from traditional food, *mg*	1123	1.1 (0-9.0)		
Dietary iron from non-traditional meats, *mg*	1123	0.5 (0-2.0)		
Heme iron, *mg*	1123	4.0 (1.2-10.6)		
Vitamin C, *mg*	1123	59.0 (15.5-162.7)		
Calcium, *mg*	1123	388 (238-610)		
Vitamin A, *μg RAE*	1123	353 (161-684)		
Tea, *mL*	1123	0 (0-500)		
Daily frequency of consumption, *n/d*				
Sea mammals	1105	0.08 (0.01-0.31)	0.117	<0.001
Game	1105	0.27 (0.07-0.77)	-0.034	0.095
Fish	1105	0.11 (0.02-0.29)	-0.014	0.735
Liver, *all species*	1105	0 (0-0.02)	0.303	0.022
Iron status and other indicators				
RBC LC-PUFA (% of total fatty acids)	1197	3.67 (1.91-5.76)	0.038	<0.001
Serum hs-C-reactive protein (mg/L)	1210	1.3 (0.4-3.4)	0.031	<0.001
Hemoglobin (g/L)	1149	128 (119-136)	0.009	<0.001
Serum soluble transferrin receptor (mg/L)	575	1.33 (1.12-1.63)	-0.287	<0.001
Serum ferritin (μg/L)	1210	24.5 (14.0-51.0)	-	-
*H. pylori* positive	1268	71.9 (69.4-74.3)^2^	-0.047	0.044

^1^Values are median (interquartile range).

^2^Values are % (95% CI).

^3^After multivariate adjustment for menopausal status, adiposity, and oral contraceptive use. Associations of <0.005 were considered significant after applying the Bonferroni adjustment.

Median intakes from carbohydrate, protein and fat (as% energy) were 45.2%, 17.3% and 29.4%, respectively. Prevalence of dietary iron inadequacy (adjusted intakes below the age-specific EAR) was much higher among pre-menopausal women (15.7-17.3%) than postmenopausal women (<1%). A small portion of the sample (4.1%) had adjusted iron intakes above the upper limit DRI (45 mg). Heme iron comprised 25.0% of total dietary iron intake for pre-menopausal women and 49.3% of total iron for postmenopausal women. Median intakes of vitamin C, vitamin A, and calcium were less than the EAR (Table 2). Tea and coffee were consumed by 43.8% and 3.7%, respectively, of women on the previous day. In order of contribution, the top five sources of iron for women 19-50 y were: TF meats (23%), baked products (13%), market food (MF) meats (13%), breads (10%) and mixed foods with meat (9%). For women over 50 y the greatest iron sources were: TF meats (44%), baked products (12%), bannock (8%), breads (7%), and MF meats (6%).

### Correlates of iron status

Descriptive statistics and associations of SF concentrations with dietary variables and biomarkers are reported in Table 2. After multivariate adjustment, frequency of sea mammal consumption was positively associated with SF. SF was positively associated with RBC% LC-PUFA, hs-CRP, and Hb concentrations, but negatively associated with sTfR. In a multivaritate-adjusted logistic model (Pseudo R^2^ = 0.19) postmenopausal status, at-risk% body fat, elevated hs-CRP, and oral contraceptive use were independently associated with reduced risks of depleted iron stores, respectively (Table 3). RBC LC-PUFA status was associated with a lower risk of depleted iron stores but only in food insecure women.

**Table 3:** **Multivariate logistic regression analysis Inuit women with depleted iron stores as the dependent variable**^1-2^

	**Multivariate odds ratio**^**3**^	**95% Confidence interval for OR**	**P-value**
Postmenopausal (1 = yes, 0 = no)	0.120	0.069 – 0.207	<0.001
% Body fat > cut-off(1 = yes, 0 = no)	0.414	0.297 – 0.578	<0.001
hs-CRP^2^ (1 = 3-10 mg/L, 0 = <3 mg/L)	0.571	0.369 – 0.884	0.012
RBC LC-PUFA,% *of total fatty acids*	0.896	0.793 – 1.012	0.068
Oral contraceptive use (1 = yes, 0 = no)	0.426	0.262 – 0.693	0.078
Food insecure (1 = yes, 0 = no)	1.37	0.98 – 1.92	0.001
Food insecure* RBC LC-PUFA	0.842	0.721 – 0.984	0.030

^1^ Women with hs-CRP > 10 mg/L and women with elevated iron stores were excluded from the model (*n* = 1062 in final model).

^2^ For highly prevalent outcomes, the odds ratios will tend to exaggerate the true relative risk.

^3^All variables presented were evaluated together in one model. The within-dwelling variance component was 0.1.

## Discussion

To our knowledge, this is the first representative survey to report prevalence estimates of anemia and depleted iron stores for Canadian Inuit women in ISR, Nunavut, and Nunatsiavut. Anemia was moderately prevalent according to the WHO classification system (20-39.9% = moderate public health problem). Half of the anemia cases could be attributed to IDA based on SF and hemoglobin, although this is likely an overestimate as functional ID was rare, even among premenopausal women (3.0%). According to Suominen et al (1998) IDA is characterized by sTfR > 3.6 mg/L, ferritin <22 μg/L, and hemoglobin <117 g/L [31]. In our population, we saw anemia and low ferritin but sTfR was not elevated. Therefore it would appear there must be other causes of the anemia observed. The IDA present is more likely explained by the pervasiveness of *H. pylori* exposure and not inadequate iron intake. This is supported by the high prevalence of dietary iron intake and high proportion of heme iron in the diet. In fact, dietary recalls are known to underestimate intake, making iron intake even more likely to be adequate [22]. *H. Pylori* infection is an inflammatory condition which may trigger overexpression of the iron regulatory protein hepcidin [32]. Therefore, discordance between adequate iron intake and iron status in the population may be a result of inflammation-induced hepcidin expression, resulting in degradation of the iron exporter ferroportin and diminished dietary iron absorption [14,15]. The mild anemia observed may be the result of chronic inflammation which can depress erythropoietin and hemoglobin synthesis [33], but does not appear to exacerbate severe IDA.

Despite numerous reports of high rates of ID among Arctic Indigenous women no studies, to the best of our knowledge, have investigated determinants of iron status for this population. Postmenopausal status was the strongest predictor of iron status, reflecting the rise in SF after menopause associated with lower iron requirements as a result of the cessation of menstruation [33]. Indeed, iron depletion was less common in postmenopausal women, whereas rates of anemia were higher (Table 1). Although postmenopausal women may have a higher burden of obesity and inflammation, the lower physiological requirements for iron and more traditional lifestyles may explain these differences observed with age. Oral contraceptive use, which decreases menstrual loss, was also associated with less risk of iron depletion. There was a trend toward a negative association between past exposure to *H. pylori* infection and SF, but this did not reach statistical significance (Table 2).

We report that obesity is associated with a lower risk of iron depletion. This finding does not agree with several observations in Caucasians and other populations [14,15,34]. Higher traditional food intake and RBC LC-PUFA status among obese Inuit could explain this contradictory relationship. Indeed, higher RBC LC-PUFA among Yu’pik Peoples in Alaska attenuates the positive relationship between obesity and CRP concentrations [35]. Median total % RBC n-3 fatty acids (5.1%; 3.1-7.4%) is higher in this population [36] than observed in a Caucasian population (4.5%; 3.6-6.0%) [37]. However, there was no significant difference between RBC LC-PUFA status among those with elevated body fat compared to those within the normal weight range in this population after controlling for age, smoking status and inflammation (CRP) (Table 4). Geographical analyses within this population reveal higher n-3 PUFA status (related to sea mammal and fish intake) among coastal communities, particularly in Nunatsiavut (6.6%; 5.4-8.3%) and the Baffin region of Nunavut (6.3%; 4.3-9.2%) [36]. Indeed, in our subset analysis (Table 4) there was a trend toward higher RBC LC-PUFA status among the obese after excluding in-land communities and the more westernized region (ISR) from the model. Clearly, more research is needed to clarify the relationship between adiposity and iron status for Inuit.

**Table 4:** Generalized linear model for RBC LC-PUFA associations with predictor variables

**Full model**^**1**^	**Coefficient**	**95% Confidence Interval**	**P-value**
% Body fat > cut-off(1 = yes, 0 = no)	-0.186	-0.475-0.103	0.208
*Predicted mean (0)*	*4.05*	*3.83-4.27*	*<0.001*
*Predicted mean (1)*	*3.86*	*3.68-4.04*	*<0.001*
Smoking status (1 = yes, 0 = no)	-0.337	-0.664 – 0.011	0.043
*Predicted mean (0)*	*4.18*	*3.91-4.45*	*<0.001*
*Predicted mean (1)*	*3.84*	*3.68-4.01*	*<0.001*
hs-CRP (1 = ≥10 mg/L, 0 = <10 mg/L)	-0.213	-0.751-0.325	0.437
*Predicted mean (0)*	*3.96*	*3.81-4.10*	*<0.001*
*Predicted mean (1)*	*3.74*	*3.22-4.26*	*<0.001*
Age (years)	0.086	0.076-0.096	<0.001
**Subset analysis of traditional communities**^**2**^			
% Body fat > cut-off(1 = yes, 0 = no)	0.246	-0.049-0.541	0.103
*Predicted mean (0)*	*4.37*	*4.16-4.57*	*<0.001*
*Predicted mean (1)*	*4.55*	*4.37-4.74*	*<0.001*
Smoking status (1 = yes, 0 = no)	-0.498	-0.828—0.168	0.003
*Predicted mean (0)*	*4.81*	*4.53-5.09*	*<0.001*
*Predicted mean (1)*	*4.31*	*4.15-4.47*	*<0.001*
hs-CRP (1 = ≥10 mg/L, 0 = <10 mg/L)	-0.307	-0.650-0.036	0.080
*Predicted mean (0)*	*4.52*	*4.36-4.67*	*<0.001*
*Predicted mean (1)*	*4.21*	*3.91-4.51*	*<0.001*
Age (years)	0.103	0.094-0.113	<0.001

^1^ All variables presented were evaluated together in one model (*n* = 1232), with predicted means adjusted for median age (41 years).

^2^ All variables presented were evaluated together in one model (*n* = 940), with predicted means adjusted for median age (41 years). The less traditional region of Inuvialuit Settlement Region and two land-locked communities in Nunavut were excluded from the model.

Frequency of sea mammal consumption was the only dietary variable associated with SF. Sea mammals are rich sources of dietary iron, but notably the fat from these mammals are an important source of n3 LC-PUFA for Inuit [38]. Among food insecure women, we observed that higher RBC % LC-PUFA was associated with reduced risk of iron depletion. Lower rates of chronic disease among Inuit has been attributed to their higher intake of LC-PUFA, through the anti-inflammatory properties of n3-fatty acids, [16]. Therefore, consumption of n3-PUFA may support iron status through down-regulation of inflammatory processes and subsequently improved dietary iron absorption. This interaction demonstrates the importance of TF intake (specifically marine mammals and fish) for iron status, especially when quality market foods are not abundant. It is therefore prudent for environmental and public health programs to support enhanced use of TF, sustainable harvesting and education about the importance of traditional foods throughout the Canadian Arctic for women, and, indeed, for the entire population. The current findings will enhance public health messaging about the value of TF in the contemporary Arctic diet and contribute to the global literature regarding the importance of TF for the health of Indigenous Peoples [39].

In addition to iron-rich foods, the traditional Inuit diet also contains few iron absorption inhibitors such as calcium and phytate (fibre) [3,40]. In this population, coffee consumption was infrequent and calcium intakes were well below the EAR. Tea consumption was common but not associated with iron status in exploratory analyses. Vitamin C intake was limited, but unlikely to greatly affect iron status given the reliance on heme iron sources. Low vitamin A intakes may also contribute to iron sequestration and limited iron availability for erythropoiesis but this was not supported by sTfR analyses in our population. Therefore dietary inhibitors are not likely to explain the pervasiveness of iron depletion observed.

Limitations of this study include a lack of repeat dietary recalls on the sample. In order to estimate usual intake of iron, within person variability from a previous Inuit dietary survey were relied upon. The coefficient of variation for iron intake may have changed over time. Also, there were no available data on serum hepcidin, TNF-alpha or related markers of chronic inflammation. Inflammation status assessed by hs-CRP likely underestimated the impact of inflammation in the population. Nutritional status biomarkers may not reflect status throughout the entire year, as only one season was assessed (late summer/early fall) and TF intakes vary with season.

## Conclusions

In conclusion, high rates of iron depletion were observed among young Canadian Inuit women, while a mild to moderate anemia problem was found across all age groups. The high prevalence of *H. pylori* exposure, together with dietary iron adequacy, suggests an inflammation-driven ID and mild anemia. Higher RBC % LC-PUFA associated with less risk of iron depletion among food insecure women, indicates that the anti-inflammatory effects of LC-PUFA may be important for iron status in this population.

## Abbreviations

CANDAT: Research orientated nutrient calculation system; CINE: Centre for Indigenous Peoples’ Nutrition and Environment; hs-CRP: High-sensitivity C-reactive protein; ID: Iron deficiency; IDA: Iron deficiency anemia; IDE: Iron deficient erythropoiesis; ISR: Inuvialuit Settlement Region; LC-PUFA: Long-chain polyunsaturated fatty acids; MF: Market food; SF: Serum ferritin; SIDE: Software for intake distribution estimation; sTfR: Soluble transferrin receptor; TF: Traditional food.

## Competing interests

The authors declare that they have no competing interests.

## Authors’ contributions

JJ was part of the team conducting the research, analyzed the data and wrote the manuscript. GE designed the study, conducted the research, and contributed significantly to data analysis and interpretation. HW provided consultation for dietary data collection and sample analyses, advice and data interpretation. HK provided consultation for dietary data collection and contributed to data interpretation and editorial advice. All authors have read and approved the final version of this manuscript.

## Pre-publication history

The pre-publication history for this paper can be accessed here:

http://www.biomedcentral.com/1471-2458/13/289/prepub
